# The Role of the CYP11B2 Promoter Polymorphism in the Diagnosis of Primary Aldosteronism

**DOI:** 10.3390/jcm9051519

**Published:** 2020-05-18

**Authors:** Łukasz Żukowski, Natalia Wawrusiewicz-Kurylonek, Piotr Szumowski, Małgorzata Mojsak, Saeid Abdelrazek, Janusz Myśliwiec

**Affiliations:** 1 Department of Nuclear Medicine, Medical University of Bialystok, M. Skłodowskiej-Curie St. 24A, 15–276 Bialystok, Poland; piotrmjs@wp.pl (P.S.); mni@o2.pl (M.M.); saeid@op.pl (S.A.); janusz.mysliwiec69@gmail.com (J.M.); 2 Department of Endocrinology, Diabetology and Internal Medicine, Medical University of Bialystok, M. Skłodowskiej-Curie St. 24A, 15–276 Bialystok, Poland; natalia.kurylonek@gmail.com

**Keywords:** primary aldosteronism, hypertension, adrenal incidentaloma, aldosterone synthase

## Abstract

Background: nowadays, primary aldosteronism (PA) is suggested to be the most frequent cause of secondary hypertension and it reaches 10% of whole hypertensive population. The CYP11B2 promoter polymorphism might cause aldosterone overproduction. The aim of this study was to establish whether the polymorphism CYP11B2 promoter has a significant impact on diagnostic of PA. Material and Methods: study group consisted of 239 hypertensive patients previously diagnosed with adrenal incidentaloma. For diagnose of PA were performed: screening test–aldosterone-renin ratio (ARR) and saline suppression test (SIT) as a confirmatory test. Genotyping was carried out by the real time PCR method. The significance of differences between the groups was evaluated through Student’s *t*-test. Results: our study revealed that genotype TT had plasma aldosterone concentration (PAC), ARR and SIT significantly higher in comparison with CC patients. The mean PAC in CC was 12.71 ng/dL vs. 20.55 ng/dL in TT patients (*p* = 0.037), which consequently gave a higher ARR in TT patients (119 vs. 44, *p* = 0.034). Mean aldosterone concentration in SIT was 2.40 ng/dL in CC patients and 9.99 ng/dL in TT patients (*p* = 0.046). Patients with CC genotype required less hypotensive drugs in comparison with TT genotype (*p* = 0.044). PA was recognized in 16 patients. Nine patients had TC genotype, six TT, and one with CC genotype. Conclusion: our study revealed predisposing TT genotype to PA. Additionally, patients with TT genotype, regardless of the PA presence, had more severe hypertension. The determination of the CYP11B2 promoter polymorphism seems to be useful in the diagnosis of PA, especially in cases where it is difficult to properly prepare patients for hormonal tests or even results of the hormonal test are incoherent.

## 1. Introduction

Nowadays, primary aldosteronism (PA) is considered to be the most frequent cause of secondary hypertension, reaching 10% of the hypertensive population [1]. Reports estimate incidence of PA for 8.5–13% in hypertensive and normokalemic individuals in whom the question of primary aldosteronism had never been raised [2]. Funder points out that there is no country where more than 1% of patients are diagnosed because of suspected PA, despite the fact that PA is present in every tenth patient with hypertension (HT) [3]. Despite a variety of antihypertensive drugs that are currently available, more than one-third of patients fail to achieve normotension. It is considered that, if we could normalize the blood pressure in all patients, even 13.5% of premature deaths could be avoided [4]. This makes diagnosis of primary aldosteronism very important. PA could be the most common identifiable, potentially curable form of hypertension. Screening tests for the PA-angiotensin-renin ratio (ARR) is highly sensitive, but with poor specificity. False positive cases need to be identified in order to avoid unnecessary invasive procedures. Confirmatory tests, such as saline infusion (SIT), should principally exhibit a high negative predictive value so false positives selected by ARR can be eliminated. Indeed, approximately 40% cases with a positive ARR actually display a plasma aldosterone concentration (PAC) that is adequately reduced in suppression test [5]. Screening test and confirmatory tests have some limitation. A washout of all interfering antihypertensive medications is feasible in patients with mild hypertension but is potentially problematic in others. The presence of very low renin levels (for example, at PRA values of 0.1 ng/mL/h), the ARR might be elevated, even when plasma aldosterone is also low (for example, 4 ng/dL), and it is almost certainly not consistent with PA. Conversely, sodium restriction, which is recommended to hypertensive patients, might falsely raise renin levels and therefore normalize ARR due to the responsiveness of many PA to salt restriction, therefore leading to false interpretation of PA screening [6].

The CYP11B2 promoter polymorphism might cause aldosterone overproduction [7]. The polymorphism of promoter CYP11B2 occurring at −344 site of the gene and substitute thymine (T) by cytosine (C)- is located in a putative binding site for the steroidogenic factor 1 (SF-1). The published results of researches on its role in HT are incoherent. Several of these studies have shown that CYP11B2 promoter polymorphism influences serum aldosterone level [8], urinary aldosterone excretion [9], blood pressure [10], and left ventricular size and mass [11]. The aim of this study was to establish whether CYP11B2 promoter polymorphism has a significant impact on diagnostics of PA.

## 2. Material and Methods

The recruitment for the study included 267 patients that were hospitalised between 2015 and 2019 with diagnosed adrenal incidentaloma and arterial hypertension. The study was performed in Department of Endocrinology, Diabetology, and Internal Diseases Medical University of Bialystok in Poland and it was conducted with approval by the local ethic committee (no R-I-002/255/2012). The research was an independent project and it was proceeded without any support from the industry. All patients were interviewed and basic physical and biochemical tests were performed. Biochemical tests were primarily meant to exclude others causes of hypertension. Obligatory tests for all patients were: measuring free cortisol and metoksycatecholamine fractions in daily urine collection (2×), salivary cortisol at 11 p.m., serum cortisol at 11 p.m., 1 mg dexamethasone suppression test. Table 1 shows the results.

Appropriate diagnostics was made in the case of suspected secondary hypertension. Twenty-eight patients were excluded from further study: six patients were diagnosed with pheochromocytoma, nine with hypercortisolism (one ACTH—depend Cushing syndrome, eight subclinical Cushing syndrome), one reno-vascular hypertension, one lymphatic leukemia, four hyperthyroidism, two hypothyroidism, four pituitary tumours, and one aortic stenosis.

The study had two parts. The first part, 20 mL of blood were collected for genetic testing and stored frozen at −90 °C. DNA was extracted from the peripheral blood leukocytes while using a classical salting out method. The SNP rs1799998 in the CYP11B2 gene was genotyped by TaqMan SNP genotyping assay (Thermo Fisher Scientific, Waltham, MA, USA). Details of reported single nucleotide polymorphism (SNP) may be found at the dbSNP website (http://www.ncbi.nlm.nih.gov/SNP/) under their respective accession numbers. It should be mentioned that ready to use fluorogenic TaqMan assays rs1799998 (C_8896484_10) were used for studied polymorphism. The reactions were carried out in a 7900HT Fast Real-Time PCR System (Applied Biosystems, Foster City, CA, USA) under the following conditions: 10 min. at 95 °C for starting AmpliTaq Gold activity, 40 cycles of 95 °C for 15 s, and 60 °C for 1 min. As a negative control, we used a sample without template. The negative control was helpful for measuring any false positive signal that is caused by contamination. SNP was analysed in duplicate. In the second part, dynamical tests of renin-angiotensin aldosterone system were performed: calculation of aldosterone-renin ratio (ARR) from plasma aldosterone concentration (PAC) and plasma renin activity (PRA) after 2 h orthostatic position. The next day, the confirmatory test was performed—saline suppression tests (SIT). This test was performed in all patient independently from result of ARR. The salt suppression test was based on measuring PAC after two liters of 0.9% sodium chloride infusion. The deficiency of potassium was corrected. For at least four weeks before hormonal tests diuretics and spironolactone were withdrawn and for at least two weeks β-adrenergic blockers, clonidine, methyldopa, dihydropiridine calcium channel antagonists, angiotensin-converting enzyme inhibitors, angiotensin receptor blockers, and non-steroidal anti-inflammatory drugs were also withdrawn. PAC was determined by the radioimmunoassay method with a RIAZENco kit (ZenTech, Liège, Belgium). The coefficient of variation (CV) was 5.3%, sensitivity 1.4 pg/ml, and specificity 100%. PRA was assayed by the radioimmunoassay method with the use of a REN-CT2 (Radim Deutschland GmbH, Freiburg, Germany). The incubation period of renin and angiotensinogen to generate angiotensin I for this assay was 90 min. For average PRA, a 1.6 ng/mL/h intra-assay precision CV was 10.0 and inter-assay precision CV was 5.6, sensitivity 0.018 ng/mL, and specificity 100%.

Statistical analyses were performed using STATISTICA 10 (StatSoft Polska, Krakow, Poland) and STATA 12 (StataCorp, College Station, TX, USA) software. Normality data distribution was checked by the Shapiro–Wilk test. The significance of differences between the groups was evaluated through Student’s *t*-test.

## 3. Results

The investigated group consist 132 (87 women, 45 men) heterozygous patients, i.e. with cytosine and thymine in the −344 region of the aldosterone synthase promoter (CT), 61 (41 women, 20 men) homozygote patients with cytosine (CC), and 46 (28 women, 18 men) homozygote patients with thymine (TT). The mean age was: 58.40 years (SD: 10.61) for patients with CT genotype, 60.27 years (SD: 8.16) for patients with CC genotype, 65.35 years (SD: 11.11) for patients with TT genotype. There were no statistically significant differences between groups in terms of age and sex. The duration of hypertension was also not statistically different in each group.

**Figure 1:**
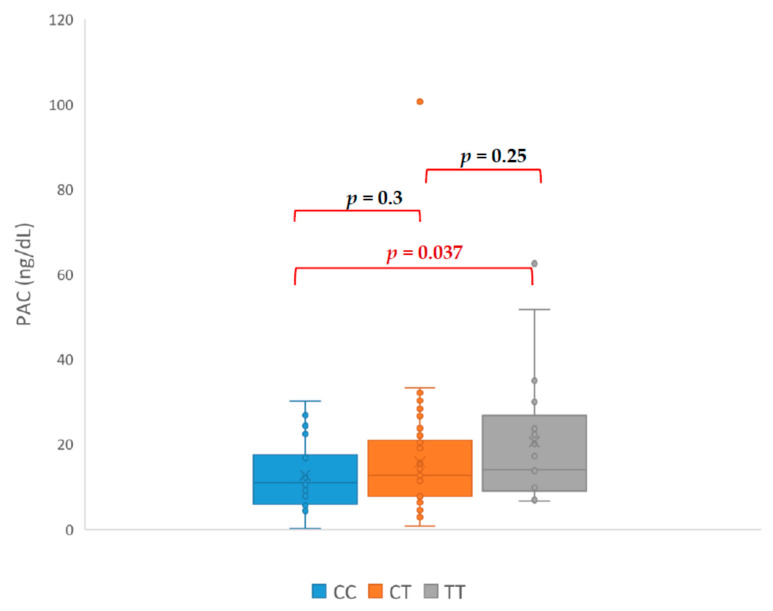
Differences in aldosterone concentrations in upright test (PAC—plasma aldosterone concentration).

**Figure 2:**
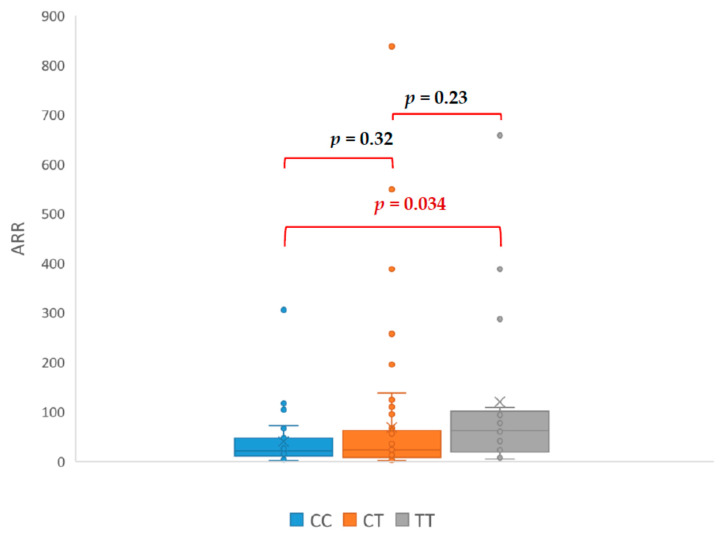
Differences in aldosterone-renin ratio (ARR—aldosterone-renin ratio).

**Figure 3:**
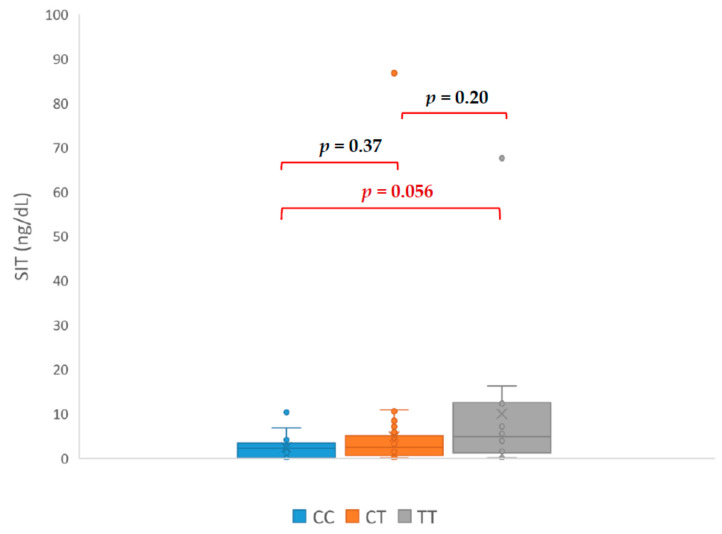
Differences in aldosterone concentrations in saline infusion suppression test (SIT—saline infusion test).

**Figure 4:**
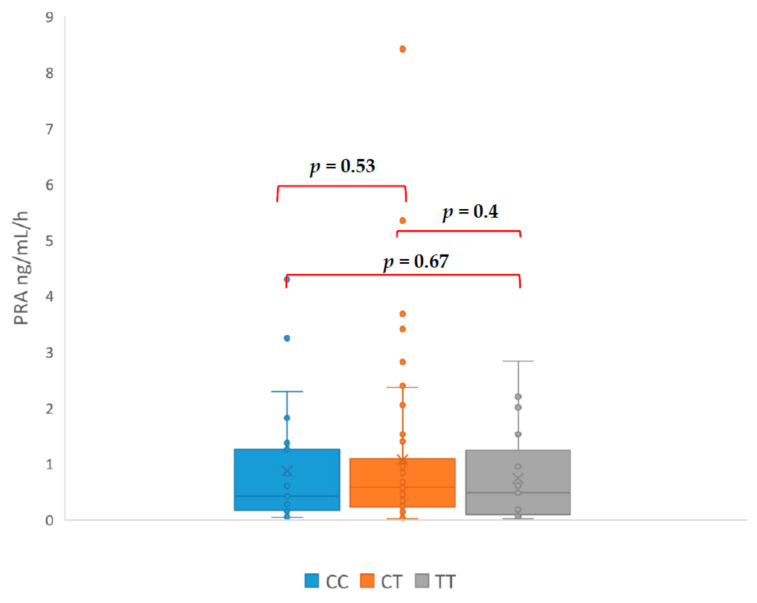
Differences in plasma renin activity (PRA—plasma renin activity).

Our study revealed that patients with genotype TT had PAC (Figure 1), ARR (Figure 2), and SIT (Figure 3) significantly higher in comparison with patients with CC genotype. The mean PAC in patients with CC genotype was 12.71 ng/dL vs. 20.55 ng/dL in patients with TT genotype (*p* = 0.037) what consequently made a higher ARR in patients with TT genotype (119 vs. 44, *p* = 0.034). Mean aldosterone concentration in SIT was 2.40 ng/dL in patients with CC genotype and 9.99 ng/dL in patients with TT genotype (*p* = 0.046). It is interesting that none of the genotypes had a significant effect on the PRA value (Figure 4). The mean PRA for CC, CT, and TT was, respectively, 0.86 vs. 1.07 vs. 0.74 ng/mL/h.

The clinical outcomes revealed that patients with CC genotype required less hypotensive drugs (Table 2) to control hypertension in comparison with TT genotype (2.361 vs. 3.080).

Following the diagnostic criteria for PA of Endocrine Society guidelines, PA was recognized in 16 patients (Table 3), which accounted for 6.7% of all cases. Nine patients had TC genotype, six TT genotype, and one CC genotype. Patient with PA and CC genotype only needed one drug to maintain normal blood pressure.

## 4. Discussion

The role of polymorphism in the RAA system is controversial. Davies et al. demonstrated a relationship with the amount of dihydrotestosterone (THA) metabolite that is excreted in the urine. TT homozygotes and heterozygotes THA have more elevated than homozygotes CC (12.4 vs. 14.6 vs. 88; *p* = 0.05). Similar results of serum aldosterone levels were received by the Hautanena et al. [12] and Paillard et al. [13]. The Japanese population has been reported to have a lesser occurrence of C allele in low renin hypertension when compared to patients with normal or high renin concentrations [14]. In the study conducted by Lim et al., a significantly higher incidence of T allele and elevated ARR in individuals with HT was found [15]. Haplotype T CYP11B2 was associated with increased aldosterone metabolite excretion and HT associated with an elevated ARR [16]. On the other hand, allele C predisposed to HT [17,18], but in one publication it was associated with the increase of ARR and in the other with ARR decrease. In another study, higher levels of aldosterone were observed in patients with CC genotype as compared to individuals with TT homozygotes [19]. Pojoga et al. found the relationship between genotype CC and C allele and elevated levels of aldosterone, but no significant difference in the mean blood pressure were noticed [19].

In our study, PAC, ARR, and SIT in the TT were significantly higher than in the CC, which suggest predisposing TT genotype to PA. Additionally, all patients with TT genotype required more antihypertensives medications to control blood pressure. Among patients with confirmed PA, only one presented CC genotype and the rest had TC (nine patients) or TT (six patients) genotype. Patient with CC genotype only required β- blocker to control blood pressure, which is unlikely for PA. The CC genotype appears to be a protective factor against the development of hypertension. This could be consistent with the results of Wang et al. research on the preoperative risk factors for persistent hypertension after successful adrenalectomy. The main determinants of surgical cure in patients with primary aldosteronism were a duration of hypertension less than five years, number of antihypertensive medications ≤2, preoperative response to spironolactone, the presence of adenoma, and the TT genotype of the CYP11B2 gene [20]. Brand et al. showed a better response to the use of AT1 antagonist in the reduction of HT in patients with T allele. Association of response to HT treatment with CYP11B2 polymorphism was also demonstrated in the SILVHIA (Swedish Irbesartan Left Ventricular Hypertrophy Investigation Versus Atenolol) study. Patients with TT genotype gained greater benefit from using AT1 antagonists than β- blockers. Carriers of the T allele also had better antihypertensive effect after taking ACE inhibitors [21].

Interesting among the results is the lack of a statistically significant difference in PRA between patients with the CC and TT genotype, although such a difference occurred with respect to PAC, ARR, and SIT. This might explain the frequent ambiguity of the results of hormonal tests used in the diagnosis of PA. Because the ARR is mathematically highly dependent on renin publications show wide range cut-off points for ARR, the usual range for ARR varies between 20 and 40. Values for aldosterone concentration in SIT between 5 and 10 ng/dL are indeterminate, although a cut-off of 6.8 ng/dL has been found to offer the best trade-off between sensitivity and specificity. This might indicate that studied polymorphism causes an increase aldosterone concentration without RAA feedback. A constant, slight overproduction of aldosterone without renin suppression might explain the mechanism of hypertension in patients with TT genotype. This mechanism of hypertension is especially possible in patients with adrenal tumour because of increased adrenocortical cellularity.

In summary: the determination of the CYP11B2 promoter polymorphism seems to be useful in the diagnosis of PA, especially in cases where it is difficult to properly prepare patients for hormonal tests or even results of the hormonal test are incoherent.

## Figures and Tables

**Table 1:** Results of hormonal tests performed to exclude hypercortisolism and pheochromocytoma.

	Cortisol in Saliva at 11 p.m.	Cortisol in Serum at 11 p.m.	Dexamethasone Suppression Test	Urinary Free Cortisol nmol/24 h	Urinary Metanephrine µg/24 h	Urinary Normetanephrine µg/24 h
Mean	0.37	2.62	1.39	49.94	110.95	345.94
SD	0.40	1.60	1.08	38.05	108.17	177.52
Ref. range	<1.2	<1.8	<1.8	<124.2	<350	<600

**Table 2:** Mean antihypertensives drug.

Mean Antyhypertensives Drugs		
**CC**	**TT**	***p***
2.361	3.080	0.044 *
**TC**	**TT**	***p***
2.703	3.080	0.22
**TC**	**CC**	***p***
2.703	2.361	0.198

* statistically significant.

**Table 3:** Characteristics of patients with primary aldosteronism.

Initials	HT Drugs Amount	Genotyp	PRA ng/mL/h	PAC (ng/dL)	ARR	SIT (ng/dL)
CA	4	TC	0.03	39.8	1300	17.8
DM	3	TC	0.12	100.6	838	86.8
LR	2	TC	0.04	21.99	549	10.56
KA	5	TT	0.09	34.97	388	13.09
PT	4	TT	0.06	17.21	287	16.24
WE	3	TC	0.1	27.5	275	8.52
KT	1	CC	0.15	17.5	117	10.3
KG	4	TC	0.03	26.61	95	8.7
MJ	5	TT	0.67	62.5	93.3	67.6
ŁR	3	TC	0.45	29.06	63	8.41
AJ	3	TT	0.48	29.93	62	6.33
DH	3	TC	0.7	23.7	60	7.07
PI	4	TT	0.5	28	56	6.6
KP	3	TC	0.4	16.76	41.9	9.21
IM	3	TT	0.5	20.68	41	16.39
TS	3	TC	0.84	30.25	36	10.91

HT—hypertension, PRA—plasma renin activity, PAC—plasma aldosterone concentration, ARR—aldosterone-renin ratio, SIT—aldosterone in saline infusion test.
